# Extensive Involvement of Alternative Polyadenylation in Single-Nucleus Neurons

**DOI:** 10.3390/genes11060709

**Published:** 2020-06-26

**Authors:** Ying Wang, Weixing Feng, Siwen Xu, Bo He

**Affiliations:** Institute of Intelligent System and Bioinformatics, College of Automation, Harbin Engineering University, Harbin 150001, China; wangy@hrbeu.edu.cn (Y.W.); fengweixing@hrbeu.edu.cn (W.F.); xusiwen@hrbeu.edu.cn (S.X.)

**Keywords:** alternative polyadenylation, neuronal heterogeneity, snRNA-seq, RBP binding motif, neuronal populations, excitatory neurons, inhibitory neurons, APA regulation

## Abstract

Cleavage and polyadenylation are essential processes that can impact many aspects of mRNA fate. Most eukaryotic genes have alternative polyadenylation (APA) events. While the heterogeneity of mRNA polyadenylation isoform choice has been studied in specific tissues, less attention has been paid to the neuronal heterogeneity of APA selection at single-nucleus resolution. APA is highly controlled during development and neuronal activation, however, to what extent APA events vary in a specific neuronal cell population and the regulatory mechanisms are still unclear. In this paper, we investigated dynamic APA usage in different cell types using snRNA-seq data of 1424 human brain cells generated by single-cell 3′ RNA sequencing. We found that distal APA sites are not only favored by global neuronal cells, but that their usage also varies between the principal types of neuronal cell populations (excitatory neurons and inhibitory neurons). A motif analysis and a gene functional analysis indicated the enrichment of RNA-binding protein (RBP) binding sites and neuronal functions for the set of genes with neuron-enhanced distal PAS usage. Our results revealed the extensive involvement of APA regulation in neuronal populations at the single-nucleus level, providing new insights into roles for APA in specific neuronal cell populations, as well as utility in future functional studies.

## 1. Introduction

Cleavage and polyadenylation (C/P) is an essential process of almost all eukaryotic mRNAs, which is coupled to the determination of 3’ ends and synthesis of poly(A) tail. C/P is defined by a set of sequence motifs around the poly(A) site (PAS). Over two-thirds of human genes have multiple PASs, also known as alternative polyadenylation (APA), leading to different transcript isoforms with distinct 3’ untranslated region (3’ UTR) and/or their coding sequence (CDS) [1,2]. Since some cis-regulatory elements for posttranscriptional regulation are located in 3’ UTR, such as binding sites for microRNAs (miRNAs) and RNA binding proteins (RBPs), many aspects of mRNA fate were impacted, including mRNA and protein localization, mRNA stability and translation [3]. It has also been implicated that APA is highly involved in the regulation of mRNA metabolism, protein diversity, gene expression, cellular functions and development of both normal cells and cancer cells [4,5,6,7]. Although evidence suggesting that genome-wide APA changes were broadly observed in specific tissues, different cell types and neuronal activation [8,9,10], it is largely unknown to what extent APA regulation varies between cell subpopulations of a specific cell type. In addition, the regulation mechanisms of APA have not been fully understood.

The diverse landscape of APA has emerged as new evidence for their important modulatory roles under different biological conditions. The APA phenomenon was originally reported in early studies, demonstrating that changes in APA usage can lead differentially post-transcriptional regulation in a tissue/cell type-specific or developmentally specific manner [11]. This has been shown in many recent studies. For example, proximal PASs were generally preferred in cells of the testis, whereas neuronal cells have the opposite trend, favoring distal PASs [12,13]; a global 3’ UTR shortening caused by APA was found in proliferating T cells of humans and mice [14]; differential APA sites formation occurs during mouse embryonic development [15,16]. Moreover, a large number of specific signals significantly impacted by APA have been observed, such as neuronal activities. Different APA isoforms show distinct functions in neurons. For instance, the distal APA isoforms of BDNF is localized to dendrites and translated upon the neuronal activity, but the short isoforms are restricted to somata [17,18]; Memo1 transcripts shifted from selecting a proximal APA isoform to a distal APA isoform during excitatory neurons’ differentiation [19].

During past few years, a growing number of APA profiles have been examined across multiple tissues for mammal species, using next-generation sequencing methods and technologies to capture the 3’-end of transcripts [20,21,22]. However, these NGS-based methods generate bulk genome or transcriptome data, which cannot provide a high-resolution view of cell-to-cell variation. Recently, single-nucleus RNA sequencing (snRNA-seq) technology has been increasingly used to investigate cellular heterogeneity because of high throughput and low technical noise. The study of the variability in APA selection at the single-nucleus level has only just started. Single-nucleus transcriptomics can provide important insights into APA regulation in specific cell types by capturing the 3’ end using single-cell 3′ RNA sequencing, i.e., 10× genomics, however, this information has rarely been used for investigating APA within a specific cell type, such as neurons.

To obtain insights into the comprehensive regulation of APA in the human brain, more specifically, in neuronal populations, we characterized the APA usage variability in neuronal cells using the paired-end snRNA-seq data of human brain cells generated from 10× Genomics Single Cell 3′ library prep, and a computational model for identifying the polyadenylation sites (PAS). A general trend was observed that brain cells generally prefer to express distal APA isoforms. We showed that APA isoform percent usage for the same gene is variable between two major types of neuronal populations. A motif analysis for RBP binding sites provided the explanations for potential mechanisms of APA regulation. We further demonstrated that the gene set with differential APA selection was significantly enriched in neuronal functions, which provided evidence for important roles for APA regulation in single neuronal cell type resolution.

## 2. Materials and Methods 

### 2.1. Sample Preparation and Single-Nucleus Sequencing

For single-nucleus sample preparation, the human brain tissues were obtained post-mortem from amygdala, which individual aged >18 years, no head injury at time of death, lack of developmental disorder, no recent cerebral stroke, no history of other psychiatric or neurological disorders, no history of intravenous or polydrug abuse, negative screen for AIDS and hepatitis B/C, and postmortem interval within 48 h. Small pieces of brain tissues in 500 μL chilled Nuclei EZ Lysis Buffer (Sigma-Aldrich, St. Louis, MI, USA) were homogenized with the pestle in a 1.5 mL microfuge tube, following Frankenstein protocol (https://community.10×genomics.com/t5/Customer-Developed-Protocols/ct-p/customer-protocols). Resulting nuclei suspension was immediately sent to flow core for single nuclei sorting by a FACS fusion sorter to collect 17K nuclei directly into the RT reaction mix (subtracting RT enzyme). We brought up and immediately measured the volume to 90 μL, and then added 10 μL RT enzyme for emulsion.

A single nucleus 3’ RNA-seq experiment is conducted using the Chromium single-cell system (10× Genomics, Inc., Pleasanton, CA, USA) and the NovaSeq 6000 sequencer (Illumina, Inc., San Diego, CA, USA). The single cell suspension was first counted on the Countess II FL for cell number, cell viability, and cell size. Depending on the quality of the initial cell suspension, the single cell preparation includes re-suspension, centrifugation, and filtration, to remove cell debris, dead cells, and cell aggregates. An appropriate number of cells were loaded on a multiple-channel micro-fluidics chip of the Chromium Single Cell Instrument (10× Genomics) with a targeted cell recovery of 10,000. Single cell gel beads in emulsion containing barcoded oligonucleotides and reverse transcriptase reagents were generated with the 3’v2 single cell reagent kit (10× Genomics). Following cell capture and cell lysis, cDNA was synthesized and amplified. An Illumina sequencing library was then prepared with the amplified cDNA. The resulting library was sequenced using a custom program on an Illumina NovaSeq 6000 sequencer. The sample (ID: AMG-SU234) was sequenced with the recommended cycles (26 bp for R1: barcode and UMI; 91 bp for R2: cDNA). In order to capture the 3’ termination sequences and accurately detect the PAS, a second sequencing run was performed for the same library (sample ID: Chrm_039_AMG-SU234), using more cycles than the recommended number, generating 2 × 150 bp paired-end constructs (R1 and R2). R1 contains the 16 bp 10× Barcode, 10 bp UMI, 30 bp poly(dT) primer sequence, and poly(A)-spanning reads; R2 contains the sequence of cDNA (Figure S1). 

### 2.2. snRNA-Seq Data Analysis

The processing of raw sequence data was performed using CellRanger v2.1.0 (10× Genomics). Briefly, CellRanger generated the FASTQ files and then aligned them to the human reference genome GRCh38 with RNAseq aligner STAR. The filtered gene-cell barcode matrices and BAM alignment files output from CellRanger were used for the downstream analysis (R 3.4.4). The initial cells were filtered and clustered using the ‘Seurat v2.3’ R package [23]. The cell type was assigned for each individual cell and cell cluster using the ‘SingleR’ R package. The visualization of each cell clustering process such as t-SNE plot was performed using R.

For human data, all initial 1424 cells were used for further analysis. Seven cell clusters were initially identified and then six different cell types including neurons (excitatory and inhibitory neurons) were classified based on the marker genes obtained from Tasic et al., 2016 [24].

### 2.3. PAS Identification

To exclude the low-quality data, a series of QC was required. Barcode, UMI and Poly(dT) primer were trimmed from poly(A)-spanning reads (R1). Trimmed reads were filtered based on their sequence length (Minimum length is 12 bp). Read trimming and length filtering were processed in a paired-end manner to be prepared for the alignment. The good quality reads were aligned to the GRCh38.p12 reference genome using STAR. Poly(A)-spanning reads (R1 from the second sequencing run) and insert cDNA reads (R2 from the first sequencing run) were aligned in paired-end and single-end mode, separately. Only uniquely mapped reads were used for further analysis. To accurately identify the original number of transcripts, PCR duplicates were removed using UMI-tools [25]. Protein coding genes were selected to annotate the 3’ positions of processed reads using bedtools v2.17. 

To identify the poly(A) sites (PAS), at the bulk cell level, a one-dimensional Gaussian mixture model (GMM) was used to model the 3′ end of snRNA-seq reads. A Gaussian mixture model is parameterized by two types of values; the mixture component weights or probabilities and the component means and variances. For a sample of *n* independent observations $x=\left\{x_{1},x_{2},\ldots ,x_{n}\right\}$, the distribution is specified by the following probability density function:

(1) $$p\left(x\right)=\sum_{i=1}^{K}\emptyset_{i}N(x|\mu_{i},\sigma_{i}),$$

(2) $$N\left(x|\mu_{i},\sigma_{i}\right)=\frac{1}{\sigma_{i}\sqrt{2\pi }}\exp\left(-\frac{{\left(x-\mu_{i}\right)}^{2}}{2\sigma_{i}{}^{2}}\right)$$

where K is the number of components; $\emptyset_{i}$ is the weight for the ith component, with the constraint that $\emptyset_{i}>0$, $\sum_{i=1}^{K}\emptyset_{i}=1$ so that the total probability distribution normalizes to 1. $N\left(x|\mu_{i},\sigma_{i}\right)\;$ is the i th component density for observation x, which is a Gaussian distribution with a mean of $\;\mu_{i}$ and variance of $\sigma_{i}$. The mean vector $\mu_{i}$ is the center of ith distribution, representing a candidate PAS. These unknown parameters ($\emptyset_{i},\mu_{i},\;\sigma_{i})$ for each component were estimated using the ‘clustCombi’ function of the ‘Mclust’ package in R [26,27]. 

Only genes with more than 100 reads and non-overlap with other genes at the chromosomal coordinates were used. To reduce the false-positive derived from ‘internal priming’, the genomic sequence in a ±10 nt window around the candidate PAS was examined. If the sequence has six continuous As/Ts, or more than 70% As/Ts, it is considered as an internal priming site, which should be filtered out. However, if an internal priming site was supported by a poly(A) signal (AAUAAA or 11 variants) in 40 nt upstream of the candidate PAS, it is considered as a real poly(A) site. 

Because a lot of continuous 5’ Ts would lead to the deterioration of sequencing quality, the read quality of insert reads (R2) is much higher than that of Poly(A)-spanning reads (R1); we use R1 for PAS identification, R2 for PAS usage quantification. PAS usage was calculated as the fraction of reads that support the PAS compared to the total reads that support the APA sites of each gene. A similar PAS identification procedure was taken for R2. To find a highly confident PAS, a series of filtering strategies was needed. A PAS identified from R2 that was supported by the PAS from R1, at least 10 reads and with the usage of 1% or more became part of the atlas. After performing the filtering criteria above, we identified 49,305 PAS in 7240 protein-coding genes, based on the bulk cell level. 

All the detected PAS were compared with the annotated PAS from PolyA_DB version 3 (http://exon.umdnj.edu/polya_db/) and GENCODE release 29 (https://www.gencodegenes.org/human/release_29.html). Considering the distance between PAS (from R2) and the 3’ termination of transcript caused by sequencing protocol, they were identical if an annotate PAS or poly(A) signal located within ~200 nt downstream of the detected PAS. We thus generated a table of alternative polyadenylation (APA) for each gene, including gene ID, PAS position and UMI (unique molecular indices) count. For downstream analysis, we only focus on the top 2 most abundant PAS (*n* = 12,770) for 6385 genes. The top 2 PAS were further defined to distal PAS and proximal PAS, separately (relative to 5’ end).

### 2.4. Differential APA Usage Testing

We mapped the PAS to two interested cell groups (excitatory neurons and inhibitory neurons), according to the information of PAS detected above from bulk cells, separately, using ‘normalmixEM’ function in R. The usage of the top 2 most abundant APA for each cell group was thus quantified. 

Fisher’s exact test was performed to evaluate whether a specific APA is differentially used in two neuron sub-groups. Odds ratio (OR) was calculated using the number of distal and proximal PAS reads in two groups. APA genes with OR > 2 and FDR < 0.05 were considered significant distal PAS isoforms up-regulated in excitatory neurons, while APA genes with OR < 0.5 and FDR < 0.05 were considered significant distal PAS isoforms down-regulated in excitatory neurons. Manual browsing to validate transcript isoforms was performed using the WashU Epigenome Browser [28] (http://epigenomegateway.wustl.edu/legacy/).

### 2.5. Motif Analysis

Sequences around the polyadenylation sites (PAS) (±100 nt) were scanned for RBP binding motifs using HOMER software from UCSD (http://homer.ucsd.edu/homer/) [29]. The searching sequence was divided into four regions relative to the PAS coordinate Pi (i = 1,2; representing the proximal and distal PAS), i.e., −100/−41, −40/−1, +1/+40 and +41/+100 nt. Known RBP binding motifs to check for enrichment were obtained from the cisBP database [30]. The portions of edPAS in excitatory neurons relative to inhibitory neurons were scanned for searching the known RBP motifs enrichment.

### 2.6. Functional Enrichment Analysis

To investigate whether the genes with significant differential APA usage in different cell groups were enriched in specific GO term and functional pathways, the genes were subject to KEGG pathway and GO enrichment analysis using clusterProfiler (Fisher’s exact test, *p*-value < 0.05) [31]. Pathway annotations and gene function (Biological Process and Molecular Function) annotations of human were retrieved from the Bioconductor package—org.Hs.eg.db.

## 3. Results

### 3.1. Evaluation of APA Based on snRNA-seq Data

To investigate APA events in the human brain based on single-nucleus RNA-seq data, we used a density-based method to identify candidate poly(A) sites, using 1424 brain cells detected from single human post-mortem brain. The single cell 3′ libraries were prepared following the standard recommendations by 10× genomics single cell protocols. Then, the resulting libraries were sequenced with not only the recommended cycles (26 × 91 bp), but also more cycles than regularly (150 PE), thus generating cDNA reads (~514 M) and poly(A) spanning reads (~99 M), separately. To achieve an accurate identification and the quantification of real poly(A) sites, we processed the snRNA-seq in paired-end mode. The poly(A) spanning reads (150 bp) were used for poly(A) site identification, and the cDNA reads (91 bp) were used for PAS usage quantification (Figure S1 and Table 1). We first needed to perform the quality control for the raw reads. Barcode, UMI and Poly(dT) primer were trimmed from the raw poly(A) spanning reads. Notably, 7.6% of reads with over 90% bases of As or Ts were removed. The trimmed reads were then aligned to HG38 using STAR and filtered by the rules that the two paired-end reads were uniquely mapped to the same chromosome. We performed UMI deduplication for the filtered reads and finally obtained around 11.6% (11.5 M) of processed poly(A) spanning reads for further analysis (Tables S1 and S2).

**Figure 1:**
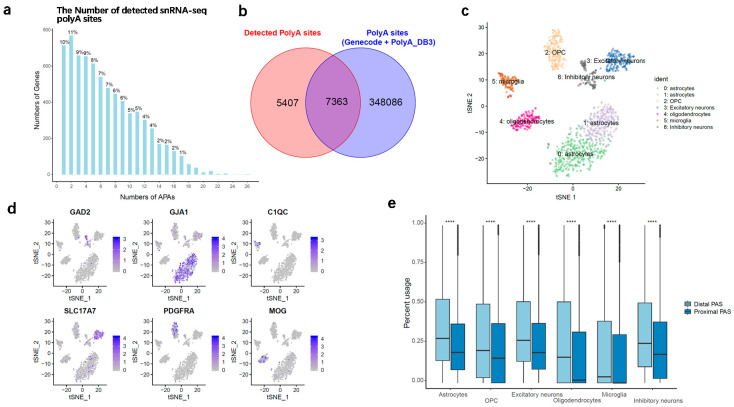
Single-nucleus alternative polyadenylation (APA) evaluation. (**a**) Distribution of poly(A) sites per gene; (**b**) Detected snRNA-seq poly(A) sites overlap with poly(A) annotation for protein coding genes; (**c**) Cell type identification; (**d**) Marker genes expression; (**e**) Distribution of percent usage of distal and proximal PAS (relative to 5’ end), which were selected from the top 2 most used APAs for each gene. **** *p*-value < 0.0001

Having established the snRNA-seq data for detecting PAS, we first asked how often genes selected more than one active PAS. As described in the methods, the 3’ most location of reads were fitted by a Gaussian mixture model (GMM), and the mean of each Gaussian density was defined as the coordinate of candidate poly(A) site. Protein coding genes, excluding overlapping genes, were used for annotation. A series of filtering criteria were taken to narrow down the highly confident PAS. To remove the false positive, internal priming events were removed from the candidate PAS. Finally, we identified 49,305 PAS covered by 7240 protein-coding genes from the pooled cells dataset. As shown in Figure 1a, around 90% of the 7240 protein-coding genes expressed more than one PAS, counting all genes with at least 10 molecules that supported a PAS. The same trend was also observed for a more conservative subset of genes, with expression in at least one-third of all cells (Figure S2). We focus on the top two most abundant PAS (*n* = 12,770) of 6385 genes for downstream analysis.

To characterize the APA usage of cell types, the top two most abundant PAS identified from snRNA-seq data were compared with genome-wide PAS annotations obtained from PolyA_DB version 3 [32] and GENCODE release 29. Not surprisingly, we found that over 57% of PAS (*n* = 7363) were overlapped with annotation, with a distance of less than 200 bp from their nearest PAS annotation (Figure 1b and Figure S3). This result showed a good match between our detected PAS and the reference PAS. We also observed that a considerable fraction of PAS identified in our dataset did not overlap annotate records; a similar phenomenon can be found by other studies [33,34]. The non-overlap set can be considered as a valuable resource for the discovery of novel PAS. Next, the top two PASs were assigned to distal PAS and proximal PAS, separately (relative to 5’ end), for testing the preference of PAS in different cell groups. Again, over 53% of these snRNA-seq PAS show consistence with PAS inferred by human brain PolyA-seq studied by Xu C et al. 2018 [35] (40% overlapping distal PAS and 13% overlapping proximal PAS, Figure S4). As shown in Figure 1c,d, cells were clustered and classified into six cell types, including astrocytes, OPC, oligodendrocytes, microglia, excitatory neurons, and inhibitory neurons, according to the information of marker genes. The number of cells, protein-coding genes and UMIs of each cell type were summarized in Table 2. As expected, a general trend was observed in our data that almost all the cell groups prefer to use distal PAS rather than proximal PAS (Figure 1e). These results were highly consistent with the findings reported by previous studies, demonstrating that cells seem to express significantly longer 3’ UTR isoforms in the central nervous system [36].

### 3.2. Cell-Type Specific APA Usage in Excitatory and Inhibitory Neurons

**Figure 2:**
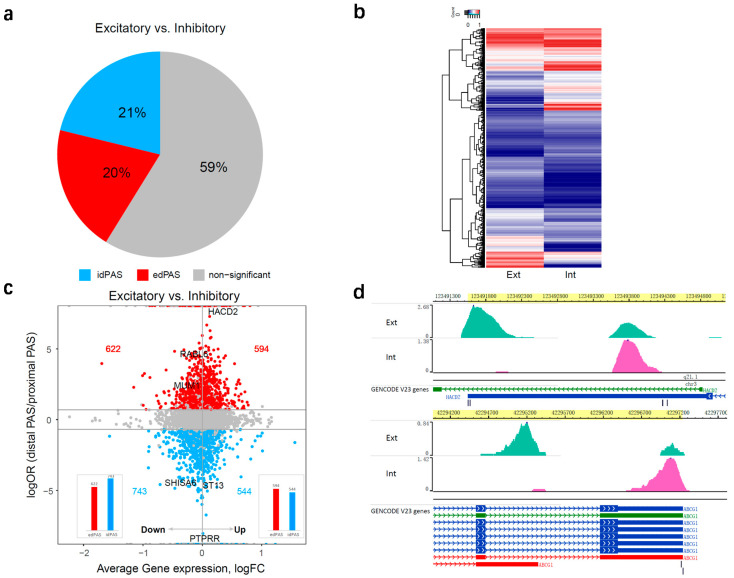
Cell-type specific APA usage in excitatory and Inhibitory neurons. (**a**) Significant changes of top 2 APAs (distal and proximal) usage in excitatory neurons, versus inhibitory neurons based on Fisher’s exact (FDR = 0.05, odd ratio less than 0.5 or more than 2). idPAS indicates inhibitory neuron-enhanced distal PAS usage; edPAS indicates excitatory neuron-enhanced distal PAS usage; (**b**) Differential percent usage of top 2 APAs between excitatory (Ext) and inhibitory (Int) neurons (FDR = 0.05). Colors represent the percentage of distal PAS and proximal PAS for 2503 genes: red indicates a high level, blue indicates a low level; (**c**) Scatterplots showing the relative (fold) change between the average gene expression level in excitatory neurons and inhibitory neurons (excitatory/inhibitory, x axis) response to the relative changes (log odds ratio) between distal PAS and proximal PAS (y axis). Red dots showing edPAS; blue dots showing idPAS; (**d**) snRNA-seq tracks for a cell-type specific APA selection gene (HACD2, ABCG1), showing evidence for different APA selection in excitatory neurons compare to inhibitory neurons. The colored regions correspond to mapped single cell 3’ RNA-seq reads from each cell group. Green indicates the reads of excitatory neurons (Ext). Pink indicates the reads of inhibitory neurons (Int).

Since the distal APA was preferred by brain cells, it is likely to play an important role in the regulation of neuronal properties. To further investigate the dynamic changes in APA usage and the regulation of APA in a different type of neurons, we selected the top two most abundant APA (*n* = 12,154), expressed in excitatory neurons and/or inhibitory neurons for comparison. Fisher’s exact test and BH corrections were performed to test the statistical significance of results. Significant differential distal APA usage was identified with FDR = 0.05, odds ratio > 2 or odds ratio < 0.5. As a result, a large number of APA switching was observed between two neuronal cell types, some of which show that distal PAS is used more than proximal PAS in excitatory neurons; these PAS were termed as excitatory neuron-enhanced distal PAS usage (edPAS); conversely, they were termed as inhibitory neuron-enhanced distal PAS usage (idPAS). The proportion of idPAS isoforms is slightly higher than that of edPAS isoforms, accounting for 21% and 20%, separately (Figure 2a, Table S3). 

Among 6077 APA genes, a total of 2503 genes were identified statistically, differentially expressing APA isoforms in excitatory and inhibitory neurons (Figure 2b). Although global profiles of gene expression were generally well correlated between the two major neuronal cell types with the Pearson correlation coefficient of 0.69 (*p*-value < 2.2 × 10^−16^, Figure S5), APA may contribute to the gene expression regulation in specific cell types, leading to the neuronal heterogeneity. To address this hypothesis, we further analyzed the relationship between relative usage of distal APA (odds ratio of distal APA events to proximal APA events in excitatory and inhibitory neurons) and the relative (fold) change between the average gene expression level in excitatory neurons and inhibitory neurons for the APA genes. Average gene expression was calculated using the log normalized gene expression measurements for two types of neurons. As shown in Figure 2c, the increase or decrease of gene expression in excitatory neurons relative to inhibitory were significantly influenced by the selection of edPAS or idPAS (*p*-value = 9.5 × 10^−4^, Chi-square test). Genes with increased expression in excitatory neurons prefer using edPAS, whereas genes with declined expression have the opposite trend, favoring idPAS. This result indicated that changes in gene expression between neuron subtypes may be explained by the distal APA site usage. Variations of distal APA site usage can also be discerned among different embryonic tissues, having a significant correlation with gene expression level, either positive or negative, coinciding with the regulation of different biological processes [37]. A global trend was observed by Chen et al. that distal PAS preference led to a global lengthening of 3’ UTRs and reduced gene expression in senescent cells [32]. This phenomenon was also observed in part of our data. For example, around 51% of the cases, in excitatory neurons where the longer APA isoform of the gene is preferred, the gene expression level is lower compared to that in inhibitory neurons. This result provides new insight into the potential role of APA in influencing gene expression in different neuron cell types. Distal APA isoform has a longer 3’ UTR region, that may serve as a platform to recruit post-transcriptional regulators, such as miRNA. This observation was consistent with the prediction of miRNA 3’ UTR targets, using a random-forest-based approach integrated by the miRWalk database [38]. Here, we observed a considerably larger proportion of miRNA gene targets in the edPAS set compared with that of the idPAS set (Figure S6). In addition, a large number of genes (~49%) were also up-regulated when the distal APA site is used, implying the existence of other potential mechanisms of APA. Thus, the changes of distal APA usage couple with the gene expression pattern in neuronal cells.

Furthermore, a cell-type specific APA selection pattern was found in this dynamic APA changing set, showing a specific selection of distal or proximal APA in excitatory neurons and inhibitory neurons. The top significant idPAS or edPAS genes can also be used to classify neuronal subpopulations, such as ST13, SHISA6, PTPRR, RABL6, MUM1 and HACD2 (Figure 2c, Figure S7). Genes undergoing differential APA selection in different neuronal cell types seem to impact neuronal activity. For example, HACD2 has two APA sites expressed in bulk cells but showing a cell-type specific selection—the distal APA site was predominant in excitatory neurons, whereas proximal APA was preferred in inhibitory neurons (Figure 2d). Previously, APA was also found to be highly tissue-specific [9]. These results identified neuronal cell-type specific APA regulation patterns, providing potential explanations for cell-type specific regulation mechanisms.

### 3.3. RBP-Binding Motif Enrichment Analysis

**Figure 3:**
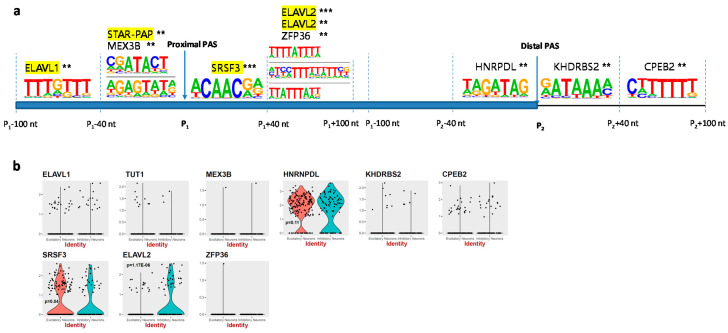
Identification of RNA-binding protein (RBP)-binding motifs around PAS. (**a**) Known RBP-binding motifs based on comparison of edPAS versus idPAS. Yellow colored RBPs were found to be involved in regulating APA (Neve J et al. RNA Biology. 2017; Camron D. Bryant et al. Genes Brain Behav.2016). * represents *p*-value is less than 0.05; ** represents *p*-value is less than 0.01; *** indicates *p*-value is less than 0.001; (**b**) Neuron specific expression of RBPs. Y axis value showing the normalized and natural log-transformed single cell expression. X axis showing the cell types, excitatory neurons colored in red and inhibitory neurons colored in green. *p*-value indicates evidence of differential expression of RBP between two groups examined using the Wilcoxon Rank Sum test.

To examine the underlying regulatory sequence motifs for the cell-type specific APA events, we performed RBP-binding protein (RBP) motif enrichment analysis based on the genes with significant differential PAS usage in two neuron sub-groups. Here, we focus on the cis-elements regulating APA sites’ usage level between excitatory neurons and inhibitory neurons. Two sets of APA sites were compared, which were edPAS versus idPAS. Six or eight hexamer frequencies in four regions around the PAS were compared, then the known RBP binding motifs were found and combined in the end, using HOMER (Figure 3a). As a result, 10 human RBP binding motifs were significantly enriched in these regions. Four RBPs were found to be involved in APA regulation in the region of around proximal PAS, including ELAVL1, STAR-PAP, SRSF3 and ELAVL2 [39,40]. Some RBPs were observed to be specifically expressed in neuron groups, such as SRSF3 (*p*-value = 0.04, Wilcoxon rank sum test) and ELAVL2 (*p*-value = 1.17 × 10^−6^, Wilcoxon rank sum test), although six identified RBPs have zero gene expression level in the majority of the cells from all the clusters (Figure 3b). Differential splicing was also observed for the gene coding of ELAVL2 [41], which were specifically expressed in inhibitory neurons in our dataset. This indicates that these sequence motifs may bind with RBPs, which in turn recruit more polyadenylation complex factors and specifically regulate the APA events. We also tested how the enrichment of RBP binding motifs for the sequences of the same regions around idPAS compare with edPAS, by exchanging the background dataset. Although the signals tested were slightly different from the previous one, some meaningful motifs are still observed, such as MSI1, MSI2 and PCBP2 (Figure S8A). PCBPs were found to regulate axonogenesis in neurodevelopment [42]. The two Musashi RBP protein family members, Musashi1 (MSI1) and Musashi2 (MSI2), have been previously observed to mediate polyadenylation [43,44]. Interestingly, MSI2 was also found to be specifically expressed in inhibitory neurons in our dataset (Figure S8B).

### 3.4. Distal APA Contributions to Neural Function

**Figure 4:**
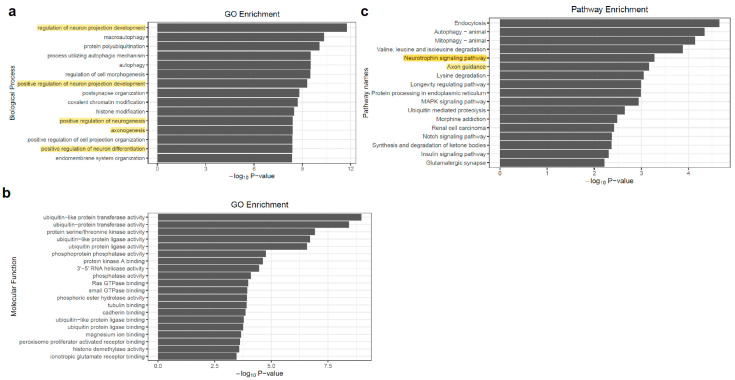
Enrichment of gene ontology annotation for the genes with significant changes of APA usage between two types of neurons. (**a**) Biological Process; (**b**) Molecular Function; (**c**) KEGG pathway.

To address the functional consequences of APA events, we tested for the enrichment of gene ontology and functional pathway annotations among the genes with differentially APA used in excitatory neurons compare to inhibitory neurons (a combined gene set with edPAS and idPAS). Not surprisingly, these genes were highly enriched for biological processes relating to neural development or neural function (Figure 4a). Among these biological processes’ terms, the highest statistical enrichment observed was “regulation of neuron projection development” (adjust *p*-value = 1.05 × 10^−8^). Other categories of strongly enriched neural related biological processes included the positive regulation of neuron differentiation (adjust *p*-value = 4.27 × 10^−7^), the positive regulation of neurogenesis (adjust *p*-value = 1.78 × 10^−6^), axonogenesis (adjust *p*-value = 1.78 × 10^−6^), and the positive regulation of neuron differentiation (adjust *p*-value = 1.78 × 10^−6^). For the molecular function terms, we observed various categories of “binding” and “activity” (Figure 4b). For the pathway enrichment, a high statistical enrichment observed was “Neurotrophin signaling pathway”, with a *p*-value of 5.37 × 10^−4^ (Figure 4c). It has been demonstrated that neurotrophin can control many aspects of neuronal development and function. Neurotrophin also regulates cell fate decisions, axon growth, dendrite growth and the expression of proteins, such as transmitter biosynthetic enzymes and neuropeptide transmitters that are essential for the normal function of neurons [45]. In summary, we observed that a set of genes with neuronal regulatory functions was preferentially subject to distal APA. 

## 4. Discussion

We have examined the use of APAs in different cell types, using a dataset comprising thousands of human brain cells, and found that APA selection can differ between specific neuronal cells. This variability was supported by statistical significance. Furthermore, the enrichment of RBP binding sites and neuronal functions were found in the set of isoforms preferring distal APA. 

To investigate the involvement of APA in human neurons on a genome-wide scale, we performed a model based poly(A) site identification and quantification approach in a single-nucleus. Polyadenylation has previously been widely examined in bulk data [6,35,46,47]. However, the dynamic APA isoform expression in single-cell or single-nucleus is beyond the scope of bulk APA research. As with other recent APA studies, we found that the utility of state-of-the-art snRNA-seq or scRNA-seq technology can help us to investigate APA usage on a transcriptom-wide scale at a high resolution level [48,49,50], e.g., cell-to-cell heterogeneity or cellular heterogeneity. A similar recent approach (BATSeq) was applied to characterize the extent of 3’ UTR choice variability in single cells [48]. We used the same sequencing protocol, that is using an oligo-dT primer containing a unique molecular identifier (UMI) and cell barcode to anchor the poly(A) tail, and obtained 3′ termination information for further APA analysis. While the sequencing protocol is similar, the strategy of data processing, identification and measurement of the APA usage was different. For example, BATSeq used a window based method to identify the PAS and assign the alignment position to the closest PAS. In this work, a density model based method was used to identify and quantify PAS. The limitation of the method in this work requires sufficient read coverage for fitting mixtures of probability distributions. When the average coverage is very low, the algorithm may fail to fit the low-coverage distribution, and further located the accurate PAS location. Considering sample size is a potential limitation of this study, the extensive APA changes observed in different cell types may still not be a global conclusion, due to individual heterogeneity. Based on these differences in PAS identification methods, we predict that approaches using high-throughput scRNA-seq or snRNA-seq data with more samples and cells will achieve greater accuracy for APA analysis. Previously, the preferential 3′ UTR extension in neural tissues has been observed [51], which is likely to be an important mechanism to control cellular functions. These distal APA isoforms leading to longer 3′ UTR length could harbor some important binding sites, recruit RNA binding complexes, and play roles in post-transcriptional regulation through cis and/or trans mechanisms. Here, we investigated the important APA events which may contribute to the distinct cellular properties of different neuronal cell types at single-nucleus resolution, and achieved considerable accuracy in PAS identification and quantification. We observed a consistent trend with previous studies that distal APA was preferentially selected by neurons.

The causal factor for the APA diversity between cell types profiled in this study remains unclear. Although we examined multiple binding sites for RBPs which involved APA regulation, the underlying mechanism is unclear. Similarly, like the complex gene regulation network, APA regulation may be determined by the sum of mediation of cis and trans contributions. The additional levels of APA regulation and their driver factors contribute to the cell-to-cell heterogeneity that will need to be studied in the future.

In summary, we found a significant diversity of APA usage between neuronal cell types. This finding provides a complementary explanation for post-transcriptional regulatory mechanisms.

## Figures and Tables

**Table 1:** Summary metrics for 10× Genomics snRNA-seq. The estimates were produced by CellRanger on raw data. Sample AMG-SU234 (Read 2) was used for quantification and main poly(A) site (PAS) analysis; sample Chrm_039_AMG-SU234 (Read 1) was used for sequencing poly(A) spanning reads and PAS identification. For detailed definitions of metrics, refer to the 10× Genomics support website, https://support.10×genomics.com/single-cell-gene-expression/software/pipelines/latest/output/summary.

	AMG-SU234	Chrm_039_AMG-SU234
Estimated Number of Cells	1424	1224
Mean Reads per Cell	361,026	78,650
Median Genes per Cell	622	162
Number of Reads	514,102,287	96,268,648
Valid Barcodes	97.4%	98.5%
Sequencing Saturation	96.3%	64.7%
Q30 Bases in Barcode	93.9%	98.7%
Q30 Bases in RNA Read	90.9%	20.3%
Q30 Bases in Sample Index	92.1%	-
Q30 Bases in UMI	93.9%	98.8%
Reads Mapped to Genome	91.8%	15.4%
Reads Mapped Confidently to Genome	88.4%	7.6%
Reads Mapped Confidently to Intergenic Regions	8.0%	1.2%
Reads Mapped Confidently to Intronic Regions	44.2%	4.3%
Reads Mapped Confidently to Exonic Regions	36.2%	2.0%
Reads Mapped Confidently to Transcriptome	33.5%	1.8%
Reads Mapped Antisense to Gene	1.4%	0.2%
Fraction Reads in Cells	64.6%	63.9%
Total Genes Detected	20,591	16,395
Median UMI Counts per Cell	791	202

**Table 2:** Number of cells, protein coding genes and UMIs per cell type in our snRNA-seq data.

Cell Type	# Cells	# Genes	# UMIs
Astrocytes	660	14,161	1,193,903
OPC	211	12,339	354,322
Excitatory neurons	177	14,180	1,175,543
Oligodendrocytes	156	10,489	162,259
Microglia	114	9915	116,823
Inhibitory neurons	106	13,035	525,975

Note: “#” represents the number of cells, genes or UMIs.

## Supplementary Materials

The following are available online at https://www.mdpi.com/2073-4425/11/6/709/s1, Figure S1: Capturing 3’ end of polyadenylated mRNA transcripts in single nucleus, Figure S2: The number of poly(A) sites per gene for 133 genes expressing in over one-third of all cells, Figure S3: Distribution of distance between detected snRNA-seq PAS and their nearest poly(A) annotations, Figure S4: Pie chart for the overlap of detected distal and proximal snRNA-seq PAS (*n* = 12,770) and PAS inferred by human brain PolyA-seq studied by Xu C, et al., 2018. Figure S5: Correlation with gene expression between excitatory neurons and inhibitory neurons, Figure S6: miRNA gene targets in edPAS set compare with that of idPAS set, Figure S7: Differentially APA usage, Figure S8: Identification of RBP-binding motifs around PAS, Table S1: Data processing polyA-spinning reads, Table S2: 3’ end annotation for processed polyA-spinning reads, Table S3: Comparison of APA usage between excitatory and inhibitory neurons.
